# Gene Polymorphisms Determining Sex Hormone-Binding Globulin Levels and Endometriosis Risk

**DOI:** 10.3390/ijms262311630

**Published:** 2025-11-30

**Authors:** Tatiana Ponomareva, Oxana Altukhova, Maria Churnosova, Inna Aristova, Evgeny Reshetnikov, Mikhail Churnosov, Irina Ponomarenko

**Affiliations:** 1 Department of Medical Biological Disciplines, Belgorod State National Research University, 308015 Belgorod, Russia; rybaarbusova@icloud.com (T.P.); churnosovamary@gmail.com (M.C.); aristova@bsuedu.ru (I.A.); reshetnikov@bsuedu.ru (E.R.); ponomarenko_i@bsuedu.ru (I.P.); 2 Department of Obstetrics and Gynecology, Belgorod State National Research University, 308015 Belgorod, Russia; altuhova_o@bsuedu.ru; 3 Obstetric Department, Belgorod Regional Clinical Hospital of St. Joasaph, 308007 Belgorod, Russia; 4 Gynecological Department, Belgorod Regional Clinical Hospital of St. Joasaph, 308007 Belgorod, Russia

**Keywords:** sex hormone-binding globulin, endometriosis, single nucleotide polymorphism, association

## Abstract

Endometriosis is a hormone-dependent disease, in the pathophysiology of which sex hormones (androgens, estrogens, etc.) are involved. The level of bioactive androgens/estrogens (in the free state) in the organism largely depends on sex hormone-binding globulin (SHBG), which binds/transports a significant portion of the androgens/estrogens of the body and, due to this, changes the amount of these hormones in a free state (bioactive), which may be important in the development of endometriosis. The study was devoted to identifying the link between the genetic determinants (single nucleotide polymorphisms [SNPs]) of SHBG (according to predating genome-wide associative studies [GWAS]) and the risk of endometriosis in the Caucasian women of Russia. The study was accomplished on a total sample of 1368 women (395 endometriosis; 973 endometriosis free [controls]). Nine loci with an impact on SHBG level in predating GWAS have been examined. The search for associations of these loci with endometriosis was carried out: both their independent effects and interlocus interactions with an in silico interpretation of the functionality/pathways in which endometriosis-related loci and strongly linked SNPs were involved have been evaluated. Polymorphic locus rs440837 (A > G) *ZBTB10* correlated with endometriosis development (recessive genetic model): the SHBG-raising genotype GG rs440837 (A > G) *ZBTB10* serves as a risk factor for the disease formation; its presence in the genotype almost doubles the risk of endometriosis (OR = 1.91; 95%CI = 1.13–2.98; p_perm_ = 0.024; power = 81.13%). The SHBG-impacts of 7 SNPs from 9 analyzed loci such as rs17496332 (A > G) *PRMT6*, rs780093 (C > T) *GCKR*, rs10454142 (T > C) *PPP1R21*, rs3779195 (T > A) *BAIAP2L1*, rs440837 (A > G) *ZBTB10*, rs7910927 (G > T) *JMJD1C*, and rs8023580 (T > C) *NR2F2* interacting with each other have been endometriosis-associated. Endometriosis-causal SNP rs440837 (A > G) *ZBTB10* and 5 proxy SNPs determine the DNA interaction in the region of 3 genes (*RP11-48B3.3*, *RP11-48B3.4*, *ZBTB10*) with 22 transcription factors and, due to this, affect the processes of development of the endocrine system, gene transcription regulation, TGF-beta signaling pathway, regulation of cell proliferation/differentiation, etc. In conclusion, the results of this study showed the endometriosis risk effect of the SHBG-impact polymorphic variants.

## 1. Introduction

Endometriosis, characterized by the appearance/proliferation of endometrial-like tissue outside the endometrium of the uterus (as a rule, the ovaries and other pelvic organs are affected), occurs in every tenth woman of reproductive age, as well as in every 2–3 women suffering from infertility [1,2,3]. The total number of women in the world diagnosed with endometriosis is as much as 176–179 million people [1]. A distinctive feature of endometriosis is a significant delay in its diagnosis, which on average is 7 years (since the appearance of the first symptoms of the disease) and requires surgical diagnostic interventions [4]. The presence of endometriosis in a woman has a significant impact on her quality of life and is associated with clinical manifestations of the disease such as dysmenorrhea, dyspareunia, chronic pelvic pain, infertility, and others [1,3,5]. Endometriosis is of extremely important socio-economic importance [4,5,6]. Thus, each woman with endometriosis, due to reduced labor productivity, loses an average of 10.8 h of work per week, which leads to significant economic costs (4 US dollars in Nigeria to 456 US dollars in Italy per woman per week) [4]. The financial costs of treating patients with endometriosis in European countries (which vary from country to country in the range of 0.8–12.5 billion Euros per year) are comparable to the cost of treating most common diseases (such as diabetes mellitus) [6].

The considerable role (up to about 50%) of hereditary factors in the formation/course (clinical manifestations) of endometriosis has been convincingly proven in previous studies performed using twin samples [7,8]. The SNP-based heritability index for endometriosis is 26% [9]. More than twenty-five GWAS have been performed for endometriosis, and even in the latest GWAS, which include very numerous patient/control samples, only a small part of the variability in endometriosis (about 5%) is described on the basis of established GWAS-impact loci [10,11,12,13,14,15,16,17,18,19,20,21,22,23,24,25,26,27,28,29,30,31,32,33,34,35,36]. For example, Sapkota and co-authors, having studied more than 200,000 DNA samples (7045 endometriosis and 191,596 controls; meta-analysis of 11 GWAS was performed) in 2017 and identified 19 GWAS-endometriosis-significant loci, explained only 5.19% of the variability in endometriosis using these loci [16]. After six years of Rahmioglu and co-authors’ work in 2023, more than 750,000 DNA samples (60,674 endometriosis and 701,926 controls; a meta-analysis of 24 GWAS was made) were studied and 42 GWAS-endometriosis-significant loci were identified that explain the same 5.01% variance of the disease [30]. Thus, despite active GWAS studies of endometriosis based on very numerous, constantly increasing samples, obtaining information about the association of the disease with an increasing number of SNPs, there was no greater clarity in understanding the genetic nature of the disease (currently known endometriosis-causal SNPs describe only about 1/20 part of its total variability and only 1/10 part of its genetic variability [16,30]), which determines the relevance of our endometriosis-oriented genetic study.

As proven in numerous clinical/experimental/review studies, endometriosis is a hormone-dependent female reproductive sphere disorder, in the pathophysiology of which various sex hormones (androgens, estrogens, etc.) are largely “involved” [37,38,39,40,41,42,43,44,45]. The level of bioactive androgens/estrogens (in a free state) in the organism (they account for only 1–2% of the hormones in the body) largely depends on SHBG [46,47,48,49]. This is due to the fact that it is SHBG that binds/transports the meaningful part (65% testosterone [50] and 38% estradiol [47]) of organism androgens/estrogens, and due to this, by changing the amount of androgens/estrogens associated with it, it can also change the amount of these hormones in a free state (bioactive) [46,47,48,49]. Such SHBG-mediated effects will directly determine the severity of the biological influences of androgens/estrogens in the organism, including those significant for endometriosis biology [37,38,39,40,41,42]. At the same time, on this topic today, the following can be stated: firstly, many GWAS data on genetic variants determining the organism SHBG level have been published [51,52,53,54,55,56,57]. Secondly, with the unquestionable pathophysiological justification for the participation of SHBG (level/expression) in the “appearance” of endometriosis, the results of specific clinical/experimental studies on this issue are ambiguous/contradictory: there are works that show the connection of SHBG with the risk of endometriosis [37,38,39], and there are works in which such correlations were not found [58]. Thirdly, the results of genetic research on this topic are few and equally ambiguous [59,60,61]. For example, in the work of Golovchenko et al., the relationship of GWAS-impact loci for the circulation level of SHBG (rs1641549 *TP53* [54], rs727428 *SHBG* [52]) was shown only in their intergenic interactions (pronounced endometriosis-significant main effects of these loci were not detected) [60]; in the work of Garitazelaia et al. (the Mendelian randomization (MR) method was used in data analysis), it was not possible to identify causal relationships between sex hormones (including SHBG) and endometriosis [61]. The above indicates the presence in the available scientific data of an obvious significant lack of experimental materials confirming (or, conversely, refuting) the relationship between the genetic determinants of SHBG and the risk of endometriosis.

The present study was devoted to identifying the link between the GWAS-validated genetic determinants of SHBG and the risk of endometriosis in Caucasian women of Russia.

## 2. Results

The clinical and anamnestic “description” of endometriosis/endometriosis free women is presented in Table 1 (these data were obtained/presented by us earlier [60]). A comparative analysis of the clinical and anamnestic indicators of “endometriosis vs. control” revealed differences in parameters such as menstrual cycle duration, parity, the anamnesis presence of medical abortions (as well as their number), infertility, surgical procedures (laparoscopy; laparotomy) on the pelvic organs, and burdened heredity (Table 1), which was the basis for “accounting” these characteristics, as well as the age of women (as confounders) in genetic assessments of “endometriosis–SNP” associations [60].

In both endometriosis/non-endometriosis subjects, the observed/expected SHBG-linked SNP genotype distribution fully meets the Hardy–Weinberg Equilibrium (HWE) standard (P_HWE_ > 0.05): in the endometriosis group, the P_HWE_ for the studied loci was in the range of 0.085 [rs440837 (A > G) *ZBTB10*]—0.763 [rs4149056 (T > C) *SLCO1B1*], and in the control group, P_HWE_—0.052 [rs3779195 (T > A) *BAIAP2L1*]—0.891 [rs780093 (C > T) *GCKR*] (Table S1).

We have identified the involvement of the polymorphic locus rs440837 (A > G) *ZBTB10* in endometriosis development (a recessive genetic model; data are shown in Table 2): the genotype GG rs440837 (A > G) *ZBTB10* serves as a risk factor for the disease formation, and its presence in the genotype almost doubles the endometriosis risk (OR = 1.91; 95%CI = 1.13–2.98; *p* = 0.023; p_perm_ = 0.024; statistical power is 81.13%).

In order to identify non-additive genetic effects that determine the risk of endometriosis, antagonistic/synergistic SNP-SNP interactions (SNP-SNP_inter_) of the studied SHBG-significant SNPs were analyzed. Four multi-level models of SNP-SNP_inter_ most valuable for disease risk were identified (Table 3): two (the significance level of the selected model was *p* = 1.25 × 10^−3^ [p_perm_ = 0.008] with a threshold value [taking into account the Bonferroni correction] equal to p_threshold_ = 1.39 × 10^−3^), three (*p* = 8.33 × 10^−6^; p_perm_ = 0.001; p_threshold_ = 5.95 × 10^−4^), four (*p* = 9.65 × 10^−8^; p_perm_ = 0.001; p_threshold_ = 3.97 × 10^−4^), and five (*p* = 1.95 × 10^−11^; p_perm_ = 0.001; p_threshold_ = 3.97 × 10^−4^) locus interactions. For almost all models at all levels (with the exception of the two-locus model), the real values of the significance level of the models significantly (by several orders of magnitude) exceeded the threshold values determined by us, taking into account the possible number of SNP-SNP_inter_ of different levels, which indicates the “reliability” of the obtained results.

Endometriosis-associated SNP-SNP_inter_ models include 7 SNPs from 9 analyzed loci, such as rs17496332 (A > G) *PRMT6*, rs780093 (C > T) *GCKR*, rs10454142 (T > C) *PPP1R21*, rs3779195 (T > A) *BAIAP2L1*, rs440837 (A > G) *ZBTB10*, rs7910927 (G > T) *JMJD1C*, and rs8023580 (T > C) *NR2F2* (Table 3). At the same time, two genetic markers—rs440837 (A > G) *ZBTB10* (it is an independent risk factor for endometriosis) and rs3779195 (T > A) *BAIAP2L1*, have been involved in the formation of the largest number of SNP-SNP_inter_ models—4 and 3 models, respectively. Attention is drawn to the fact that the five-locus model [rs8023580 *NR2F2*-rs7910927 *JMJD1C*-rs440837 *ZBTB10*-rs3779195 *BAIAP2L1*-rs780093 *GCKR*] has the most expressed effect on the occurrence of endometriosis (the risky combinations of this model were characterized by the maximum value of the Wald parameter, which was 45.02) (Table 3).

Modeling of 15 different endometriosis-associated combinations of SNP-SNP_inter_ genotypes was performed (Table S2), among which the vast majority of SNP-SNP_inter_ genotypes (12/15, 80%) were risky and only 20% (3/15) had a protective effect in the event of endometriosis. The greatest statistical significance among the identified 15 SNP-SNP_inter_ genotypes was in those such as rs440837-AG *ZBTB10*-rs3779195-TT *BAIAP2L1* (β = −0.513; *p* = 0.001), rs8023580-TC *NR2F2*-rs7910927-GT *JMJD1C*-rs440837-AG *ZBTB10*-rs3779195-TA *BAIAP2L1*-rs780093-CT *GCKR* (β = 1.698; *p* = 0.006), and rs8023580-TT *NR2F2*-rs7910927-GG *JMJD1C*-rs440837-AG *ZBTB10*-rs3779195-TA *BAIAP2L1*-rs780093-CT *GCKR* (β = 2.887; *p* = 0.008).

**Figure 1:**
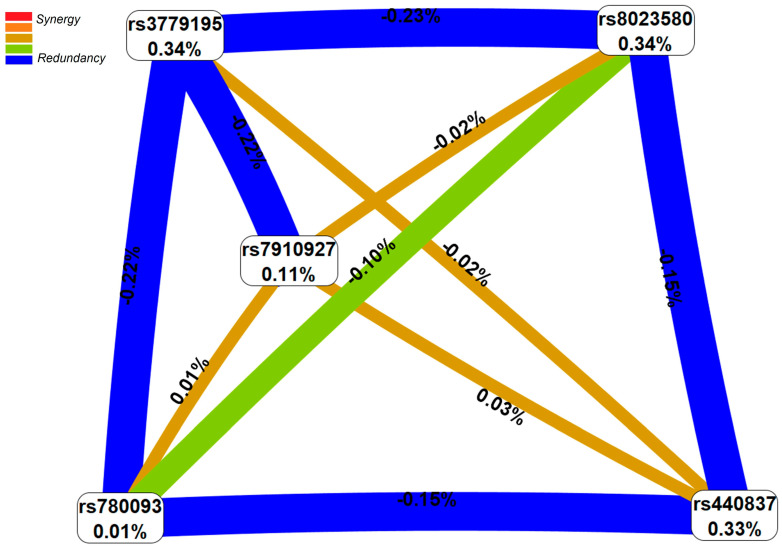
The entropy graph of the endometriosis-associated five loci model of SNP × SNP interactions (based on the MDR analysis, Wald st. = 45.02, *p* = 1.95 × 10^−11^, p_perm_ = 0.001). Positive values of entropy indicate synergistic interactions while negative values indicate redundancy. Red and orange colors denote strong and moderate synergism, respectively; brown color denotes the independent effect; green and blue colors denote moderate and strong antagonism.

**Figure 2:**
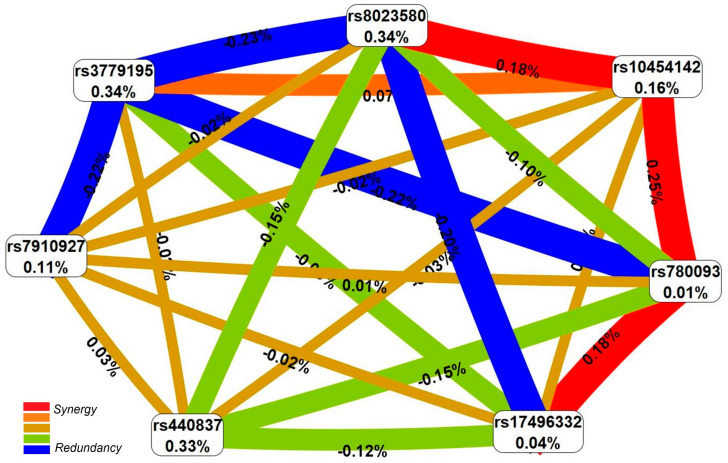
The entropy graph of the endometriosis-associated SNP × SNP interactions (based on the MDR analysis). Positive values of entropy indicate synergistic interactions while negative values indicate redundancy. Red and orange colors denote strong and moderate synergism, respectively; brown color denotes the independent effect; green and blue colors denote moderate and strong antagonism.

Paired antagonistic interactions with a contribution to trait entropy (endometriosis risk) from −0.15% to −0.23% were recorded when visualizing and evaluating the strength of the influence of the most significant endometriosis-associated five-locus SNP-SNP_inter_ model, rs8023580 *NR2F2*-rs7910927 *JMJD1C*-rs440837 *ZBTB10*-rs3779195 *BAIAP2L1*-rs780093 *GCKR* (Figure 1). For the SNP-SNP_inter_ of 7 SNPs determining susceptibility to endometriosis in all 4 models, interactions of both antagonistic (between 5 SNPs, deposit to entropy from −0.20 to −0.23%) and synergistic (between 4 SNPs, contribution to entropy from 0.18 to 0.25%) orientation were revealed (Figure 2). Three polymorphisms such as rs3779195 (T > A) *BAIAP2L1*, rs8023580 (T > C) *NR2F2*, and rs780093 (C > T) *GCKR* were involved in the largest number of the very impact-paired (with the greatest deposit to entropy) SNP-SNP_inter_ (three each).

### 2.1. Forecasted Functionality of Endometriosis-Associated Polymorphisms

At this stage of our work, firstly, we performed functional annotation of the rs440837 (A > G) *ZBTB10* locus, independently associated with endometriosis (genotype GG serves as a risk factor for the development of the disease, OR = 1.91) and 5 SNPs linked with it (r^2^ ≥ 0.80); secondly, we executed functional annotation of all seven endometriosis-associated SHBG-correlated polymorphic loci, and 115 SNPs strongly linked to them (r^2^ ≥ 0.80).

#### 2.1.1. Functional Annotation of the Endometriosis-Causal SNP rs440837 (A > G) ZBTB10

As shown by the obtained results (Table S3), the rs440837 (A > G) *ZBTB10* was localized in the region of DNA motifs serving as binding sites with 3 transcription factors (TF): Hlx1, Hoxa9, and Smad3. The endometriosis risk allele G rs440837 reduces affinity for the TF Hoxa9 (the difference in the “LOD_scores_” parameter of the G and A alleles was −1.7) and increases sensitivity to TFs Hlx1 and Smad3 (differences in the “LOD_scores_” of the G and A alleles are 1.5 and 7.5, respectively). This polymorphic locus was functionally active (located at the sites of potential promoters/enhancers, active promoters/enhancers) in the liver (the organ where SHBG is synthesized). Among the 5 polymorphisms strongly linked to rs440837 (A > G) *ZBTB10*, one locus, rs7013042, also exhibits pronounced functionality in the liver (located at the sites of promoters/enhancers, active promoters/enhancers), and all five proxy loci affect the DNA interaction with 19 TFs:rs58126885 (DMRT3, DMRT4, Pou1f1, Pou3f2), rs3893826 (CDP, CTCF, Gfi1), rs10957982 (Bbx, Mrg1::Hoxa9, Mrg, Nkx2, Sox, Tgif1), rs200988859 (DMRT5, Mef2, TCF11::MafG), rs7013042 (LXR, Myc, Sp4) (Table S4). So, rs440837 (A > G) *ZBTB10* and 5 LD loci affect the interaction of DNA with 22 TFs (DMRT3, DMRT4, DMRT5, Pou1f1, Pou3f2, CDP, Gfi1, Bbx, Mrg1::Hoxa9, Mrg, Nkx2, Sox, Mef2, Tgif1, CTCF, TCF11::MafG, LXR, Myc, Sp4, Hlx1, Hoxa9, Smad3) in the region of three adjacent genes (*RP11-48B3.3*, *RP11-48B3.4*, *ZBTB10*).

**Figure 3:**
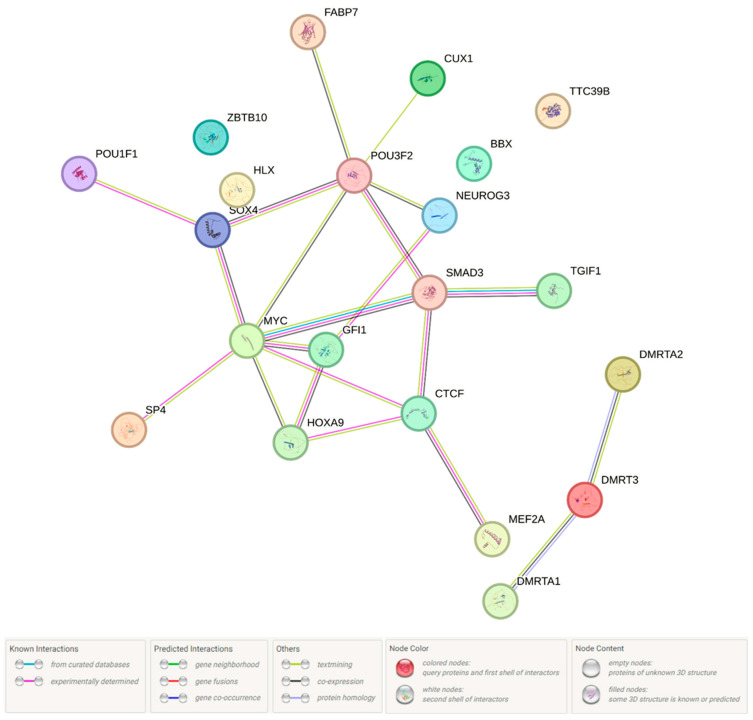
Interactions of transcription factors and proteins of the *RP11-48B3.3*, *RP11-48B3.4*, and *ZBTB10* genes linked with the endometriosis-causal SNP rs440837 (A > G) *ZBTB10* and 5 proxy SNPs (STRING program data).

**Figure 4:**
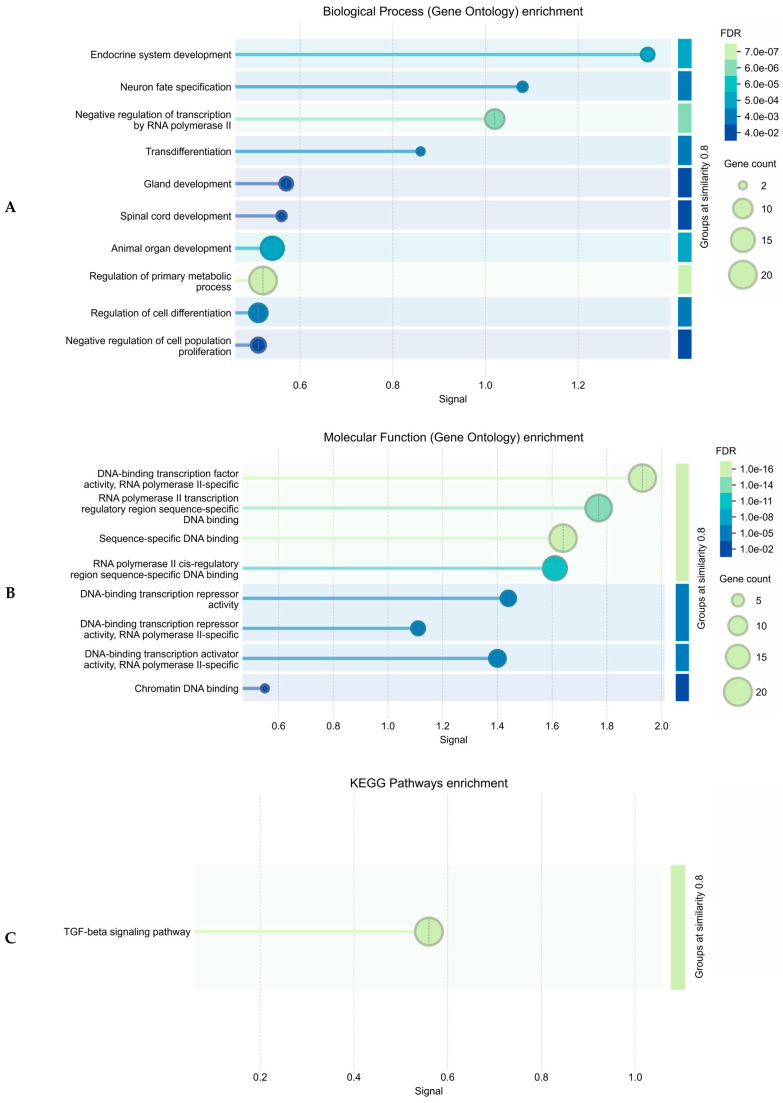
The main biological pathways linked with the endometriosis-causal SNP rs440837 (A > G) *ZBTB10* and 5 proxy SNPs: (**A**) Biological Process enrichment (Gene Ontology); (**B**) Molecular Function enrichment (Gene Ontology); (**C**) KEGG Pathway enrichment (STRING program data).

**Figure 5:**
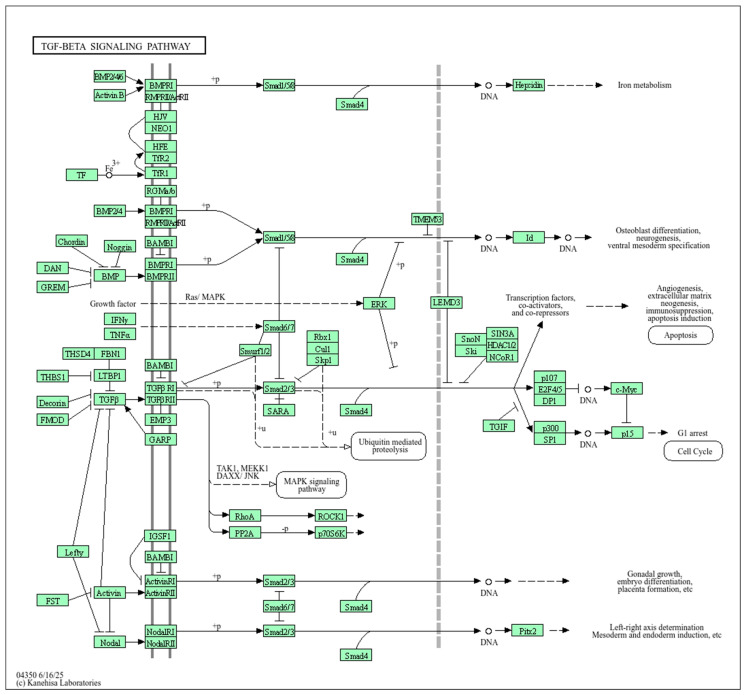
TGF-beta signaling pathway linked with the endometriosis-causal SNP rs440837 (A > G) *ZBTB10* and 5 proxy SNPs (KEGG program data, https://www.kegg.jp/kegg-bin/show_pathway?hsa04350# (accessed on 26 October 2025)).

Next, we studied the biological pathways in which these 22 TFs and protein products, determined by 3 genes in the region which contain rs440837 (A > G) *ZBTB10* and 5 proxy SNPs, have been involved (Figure 3). According to Figure 3 (it is a network of paired interactions that form TFs and proteins functionally related to rs440837 (A > G) *ZBTB10* and 5 proxy SNPs), the following TF paired interactions demonstrate the greatest contribution to endometriosis susceptibility: SMAD3-MYC (coefficient [score] of the protein interaction −0.993), TGIF1-SMAD3 (0.974), MYC-CTCF (0.763), SOX4-MYC (0.722) and CTCF-SMAD3 (0.702). The role of “hubs” in these endometriosis-significant interactions was performed by proteins such as MYC (participates in 7 pair interactions), POU3F2 (6 pair interactions) and CTCF (4 pair interactions). Endometriosis-significant protein interactions were involved in more than 15 different biological pathways—mainly in the development of the endocrine system ([GO:0035270, p_FRD_ = 0.00024] (NEUROG3, SOX4, POU3F2, SMAD3, POU1F1)) (Figure 4A), the regulation of gene transcription processes due to modulation (activation/repression) of RNA polymerase II binding to the corresponding sections of the genome, etc., ([GO:0000981, p_FRD_ = 1.01 × 10^−16^; GO:0043565, p_FRD_ = 1.34 × 10^−15^; etc.] (almost all considered 22 TFs)) (Figure 4A,B), the regulation of primary metabolic processes ([GO:0080090, p_FRD_ = 7.37 × 10^−7^] (DMRT3, SP4, NEUROG3, SOX4, GFI1, DMRTA1, BBX, TGIF1, POU3F2, SMAD3, POU1F1, HOXA9, CUX1, HLX, CTCF, DMRTA2, ZBTB10, TTC39B, MYC, MEF2A) (Figure 4A), the TGF-beta signaling pathway (Figure 4C), and the regulation of cell proliferation/differentiation ([GO:0042127, p_FRD_ = 0.0109/GO:0045595, p_FRD_ = 0.0076] (NEUROG3, SOX4, POU3F2, SMAD3, HOXA9, CUX1, HLX, DMRTA2, MYC)) (Figure 4A), etc. Interestingly, the TGF-beta signaling pathway, which is important in realizing the phenotypic effects of rs440837 (A > G) *ZBTB10* and 5 proxy SNPs (Figure 4C), is involved in endometriosis-significant mechanisms such as apoptosis, angiogenesis, extracellular matrix neogenesis, immunosuppression, cell cycle, gonadal growth, etc. (KEGG program data) (Figure 5).

Thus, the results obtained indicate the important role of endometriosis-significant protein interactions of 22 TFs and protein products of 3 genes “dependent” on the rs440837 (A > G) *ZBTB10* and 5 LD SNPs, mainly in the development of the endocrine system, regulation of gene transcription processes, the TGF-beta signaling pathway, regulation of cell proliferation/differentiation, etc.

#### 2.1.2. Functional Annotation of the Endometriosis-Associated Variants

It was found that out of 122 endometriosis-significant genetic markers (7 endometriosis-associated loci and 115 proxy variants), 118 SNPs (96.72%) have alleged regulatory influences on 13 genes in the region in in which they are located (*ZBTB10*, *RP11-48B3.4*, *RP11-327J17.3*, *RP11-327J17.2*, *PPP1R21*, *NR2F2*, *KLRAQ1*, *JMJD1C*, *GCKR*, *FOXN2*, *BRI3*, *PRMT6*, *BAIAP2L1*) (Table S4). These 118 SNPs have been localized in DNA regions considered evolutionarily conservative (5 SNPs, 4.10%), functionally active regions of the genome (promoters (10 SNPs, 8.19%) and enhancers (28 SNPs, 22.95%)), areas of hypersensitivity to the action of DNase (26 SNPs, 21.31%), and binding sites with regulatory proteins (17 SNPs, 13.93%), as well as the region of regulatory DNA motifs (110 SNPs, 90.16%) (Table S4).

We found that six endometriosis-associated loci (Table S5) and 110 LD SNPs (Table S6) were linked with the expression of 39 genes (*JMJD1C-AS1*, *JMJD1C*, *ZNF512*, *BRI3*, *TRIM54*, *TECPR1*, *STON1-GTF2A1L*, *STON1*, *SLC5A6*, *PPP1R21*, *RP11-307C18.1*, *SNX17*, *RP11-460M2.1*, *RPL7AP50*, *RP11-327J17.2*, *RP11-191L17.1*, *REEP3*, *PRMT6*, *PPM1G*, *NRBP1*, *NRBF2*, *MSH6*, *MRPL35P2*, *LMTK2*, *LHCGR*, *KRTCAP3*, *IFT172*, *AC074117.10*, *GTF2A1L*, *GPN1*, *GCKR*, *FSHR*, *FOXN2*, *FNDC4*, *C2orf16*, *BAIAP2L1*, *ATRAID*, *AC004967.7*, *ASNS*) including endometriosis-significant organs/tissues such as pituitary [4 genes] (*FOXN2*, *GTF2A1L*, *MRPL35P2*, *RP11-307C18.1*), adipose [13 genes] (*ATRAID*, *BRI3*, *GTF2A1L*, *KRTCAP3*, *MRPL35P2*, *NRBP1*, *PPM1G*, *PPP1R21*, *PRMT6*, *REEP3*, *RP11-307C18.1*, *STON1-GTF2A1L*), thyroid [21 genes] (*AC074117.10*, *ATRAID*, *BAIAP2L1*, *C2orf16*, *FNDC4*, *GCKR*, *GTF2A1L*, *IFT172*, *JMJD1C-AS1*, *KRTCAP3*, *LMTK2*, *MRPL35P2*, *PPM1G*, *PPP1R21*, *PRMT6*, *REEP3*, *RP11-307C18.1*, *RPL7AP50*, *STON1*, *TECPR1*, *ZNF512*), adrenal glands [6 genes] (*FOXN2*, *GTF2A1L*, *KRTCAP3*, *MRPL35P2*, *PRMT6*, *RP11-307C18.1*), and blood [5 genes] (*KRTCAP3*, *NRBP1*, *PRMT6*, *RP11-307C18.1*, *TECPR1*) (Tables S5 and S6).

We also found that three endometriosis-associated variants (Table S7) and 31 proxy loci (Table S8) have been involved in the alternative splicing regulation of 13 genes (*KRTCAP3*, *IFT172*, *TRIM54*, *STON1-GTF2A1L*, *BRI3*, *STON1*, *SNX17*, *PPP1R21*, *GTF2A1L*, *GPN1*, *GCKR*, *FNDC4*, *BAIAP2L1*) with an impact on endometriosis biology organs/tissues such as pituitary [3 genes] (*FNDC4*, *IFT172*, *PPP1R21*), adipose [9 genes] (*BRI3*, *FNDC4*, *GPN1*, *GTF2A1L*, *IFT172*, *PPP1R21*, *SNX17*, *STON1*, *STON1-GTF2A1L*), thyroid [4 genes] (*BRI3*, *IFT172*, *KRTCAP3*, *PPP1R21*), and adrenal glands [3 genes] (*FNDC4*, *GCKR*, *IFT172*).

Summarizing the results of the performed functional annotation of the SHBG-correlated endometriosis-associated 7 polymorphic loci and 115 LD SNPs, it can be concluded that these polymorphisms, due to their epigenetic [13 genes], eQTL [39 genes], and sQTL [13 genes] effects, can exhibit functionality with respect to 43 genes (*ZNF512*, *ZBTB10*, *TRIM54*, *TECPR1*, *STON1-GTF2A1L*, *STON1*, *SNX17*, *SLC5A6*, *RPL7AP50*, *RP11-48B3.4*, *RP11-460M2.1*, *RP11-327J17.3*, *RP11-327J17.2*, *RP11-307C18.1*, *RP11-191L17.1*, *REEP3*, *PRMT6*, *PPP1R21*, *PPM1G*, *NRBP1*, *NR2F2*, *MSH6*, *MRPL35P2*, *LMTK2*, *LHCGR*, *KRTCAP3*, *KLRAQ1*, *JMJD1C-AS1*, *BAIAP2L1*, *JMJD1C*, *GCKR*, *IFT172*, *GTF2A1L*, *GPN1*, *FSHR*, *FOXN2*, *FNDC4*, *C2orf16*, *AC074117.10*, *BRI3*, *ATRAID*, *ASNS*, *AC004967.7*).

**Figure 6:**
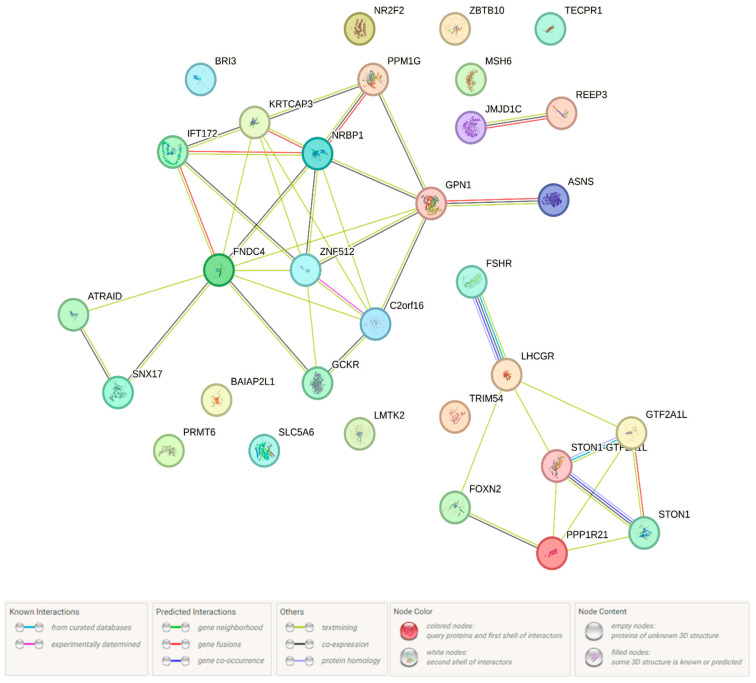
Endometriosis-associated protein interactions (the network is built in the STRING program).

Next, we performed an analysis of protein interactions linked with the above 43 genes (the STRING program was used) (Figure 6). The greatest contribution to the susceptibility to endometriosis (Figure 6; it is a network of paired interactions that form proteins functionally related to 7 endometriosis-associated loci and 115 proxy variants) came from such paired protein interactions as GTF2A1L-STON1 (coefficient [score] of the protein interaction—0.992), JMJD1C-REEP3 [0.901], C2orf16-ZNF512 [0.777], FSHR-LHCGR [0.698], KRTCAP3-NRBP1 [0.626], PPP1R21-STON1 [0.610], C2orf16-GPN1 [0.610], and KRTCAP3-ZNF512 [0.604)]. A number of paired protein interactions were characterized by impact co-expression: ATRAID-SNX17 (coefficient [score] of the protein co-expression-0.151), GPN1-PPM1G [0.146], GPN1-NRBP1 [0.138], FNDC4-GCKR [0.127], and LHCGR-FSHR [0.124]. The role of “hubs” in these endometriosis-significant interactions was performed by proteins such as FNDC4 (participates in 9 pair interactions), NRBP1 and ZNF512 (7 pair interactions each), C2orf16, GPN1, and KRTCAP3 (6 pair interactions each). These protein interactions have been performed with the participation (STRING data) of transcription factor IIA, alpha/beta subunits [CL:27093; p_FRD_ = 0.00025] (STON1-GTF2A1L; GTF2A1L; STON1), domain of unknown function DUF4515, and GPCR family 2 [CL:25526; p_FRD_ = 0.00025] (FNDC4; ZNF512; C2orf16).

## 3. Discussion

This report demonstrates the endometriosis-risk effect in Caucasian women of Russia of the SHBG-raising genotype GG rs440837 (A > G) *ZBTB10* (OR = 1.91) and shows the association of the disease with GWAS-substantial intergenic interactions for the level of SHBG and 7 functionally bulky polymorphisms within the framework of 4 multi-level models (from two to five loci) that determine (due to their assumed pronounced functionality) involvement in the endometriosis biology of many genes and TFs that affect the processes of the endocrine system development, gene transcription regulation, the TGF-beta signaling pathway, regulation of cell proliferation/differentiation, etc.

In two independent predating studies that conducted GWAS, SNP rs440837 (A > G) *ZBTB10* showed a relationship with the SHBG level [53,57]. In GWAS, Coviello et al. (all subjects were of European origin), both in the sample as a whole (21,791 individuals [meta-analysis] and 7046 individuals [validation]) and separately in the cohort of women (9390 individuals [meta-analysis] and 4509 individuals [validation]), a correlation of SNP rs440837 (A > G) *ZBTB10* with the level of SHBG was demonstrated, and at the same time, the allele A of this polymorphism marked a low level of SHBG with a more pronounced effect in women: β = −0.030 (*p* = 3 × 10^−9^) and β = −0.042 (*p* = 7 × 10^−8^), respectively [53]. GWAS by Harrison et al. on a sample of Europeans (British ancestry) also confirmed rs440837 (A > G) *ZBTB10* to be associated with comparable SHBG levels as in men (*p* = 8 × 10^−9^; 148,248 men); similarly, in postmenopausal women (*p* = 1 × 10^−12^; 104,632 women) and with high levels of SHBG in both studied groups (men and women), the allele G rs440837 (A > G) *ZBTB10* was associated as β = 0.57 and β = 1.43, respectively; it should be emphasized that based on the values of the indicator “β”, the phenotypic effect in women was more than 2.5 times higher than in men [57]. So, the data from two predating conducted GWAS, by Coviello et al. [53] and Harrison et al. [57], are completely consistent with each other and indicate that the high-level marker of SHBG was the minor allele G, and the low-level marker of SHBG is the major allele A of the rs440837 (A > G) *ZBTB10*. So, this gives us reason to conclude that the SHBG-higher genotype GG rs440837 (A > G) *ZBTB10* (GWAS data, Coviello et al. [53] and Harrison et al. [57]) correlates with a high risk of endometriosis (OR = 1.91, our data). Importantly, our previous study revealed an association of rs440837 (A > G) *ZBTB10* with uterine fibroids: the genotype GG increased the risk of uterine fibroids by almost 2 times (OR = 1.93) among women of the same population (Europeans in Russia) [62], which is fully consistent with the data of this study (the risk value of rs440837 (A > G) *ZBTB10* in the formation of both endometriosis and uterine fibroids) and allows us to consider rs440837 (A > G) *ZBTB10* as one of the potentially possible syntropic polymorphisms/genes in the development of benign proliferative diseases of the uterus. It should be noted that our assumption about the potentially possible syntropic effects of rs440837 (A > G) *ZBTB10* in the formation of uterine hyperplastic diseases (endometriosis and uterine fibroids) requires additional research and confirmation in other experimental genetic (associative/functional) studies.

It is extremely interesting that the genome region where, established by us, the endometriosis-causal SHBG-significant locus rs440837 (A > G) *ZBTB10* was located—8q21.13 (chromosome position [hg38]: 80,535,356–80,561,600)—has a very significant role in regulating the SHBG concentration in the organism: in addition to the SNP rs440837 (A > G) *ZBTB10* considered by us, according to the materials of two GWAS, four other loci were associated with the SHBG level (these loci were proxy variants of rs440837) such as rs72688090 (β = 0.038; *p* = 2 × 10^−17^ [63]; r^2^ = 0.33, D’ = 0.85), rs388922 (β = 0.47; *p* = 3 × 10^−8^ [57]; r^2^ = 0.53, D’ = 0.96), rs575452 (β = 1.31; *p* = 2 × 10^−12^ [57]; r^2^ = 0.28, D’ = 0.71), and rs117921873 (β = 1.74; *p* = 5 × 10^−10^ [57]; r^2^ = 0.26, D’ = 1.00). Along with this, strongly coupled with rs440837 (A > G) *ZBTB10* polymorphism—rs444915 (r^2^ = 0.43, D’ = 0.80), was GWAS associated with body mass index (BMI) (β = 0.011; *p* = 4 × 10^−9^ [64]). Importantly, on the one hand, BMI has inverse correlations with endometriosis (low BMI is a risk factor for the disease [65,66,67,68]), and there are works in the literature that show the association of BMI-related polymorphisms/genes (*GHRH*, *GRB14*, *LHCGR*, *KIFAP3*, *WNT4*, *CAB39L*, etc.) with endometriosis [69]; on the other hand, BMI also has inverse correlations with the SHBG level in the organism [70,71,72].

We have obtained in silico data on the serious functionality of the endometriosis-causal SNP rs440837 (A > G) *ZBTB10* and 5 proxy loci: they were localized in sites of potential promoters/enhancers, active promoters/enhancers in the DNA region of three genes such as *RP11-48B3.3*, *RP11-48B3.4*, *ZBTB10*, and affect this genome region interaction with 22 TFs. Importantly, endometriosis-causal SNP rs440837 (A > G) *ZBTB10* itself and the strongly linked variant rs7013042 manifest functional activity in the liver, which is the organ of SHBG formation [48,73]. This may be one of the pathophysiological mechanisms of the rs440837 (A > G) *ZBTB10* (with rs7013042) communicating with the SHBG level and their involvement in endometriosis pathogenesis due to this. Interestingly, the protein-product of the *ZBTB10* gene (the zinc finger and BTB domain containing 10) exhibits its effects in the nucleoplasm, and it is assumed that it participates in the regulation of transcription using RNA polymerase II (by activating its binding to a DNA sequence specific to it) [74]. There are literature data on the association of the *ZBTB10* gene with SHBG- and sex hormone-dependent tumors such as breast and ovarian cancers [74,75,76,77,78]. It is indicated that ZBTB10 inhibits the production of vascular endothelial growth factor (VEGF), its receptors of the first and second types (VEGFR1/VEGFR2), and survivin, and decreases mRNA/protein expression of specificity protein (Sp) TFs such as Sp1, Sp3, Sp4, which are responsible for the processes of tumor angiogenesis/growth/metastasis [75,76], and these biological pathways are also important for the biology of endometriosis [79]. Interestingly enough, according to our in silico data, rs7013042, strongly linked to the endometriosis-causal SNP rs440837 (A > G) *ZBTB10*, affects the “DNA-Sp4” interaction. The participation of Sp (Sp1-Sp4) in the activity regulation of the thousands genes involved in the cell proliferation/migration/survival/invasion processes has been shown [80].

The materials presented in the literature show a pronounced determination (56–58%) of the concentration of circulating SHBG by hereditary factors [46,81]. Nine polymorphisms studied in our work have a significant effect on the content of SHBG in the female organism (they account for 8.4% of the variability in the SHBG level) [53], seven of which (according to our results) determine the predisposition to endometriosis in Caucasian women of Russia. Interestingly, previously we showed an association with breast cancer risk in 8 out of 9 SNPs and the identical loci panel in the same population [78], what may indicate the “universal” role of GWAS-important genetic determinants of SHBG in the development of female reproductive sphere hormone-dependent diseases (endometriosis, breast cancer) in the population of Europeans in Russia. Similarly, the association of loci from the list of SNPs examined by us with breast cancer was demonstrated in the study by Dimou et al. (the Mendelian randomization method was used and postmenopausal women were considered) [82].

The literature materials about the link of SHBG level/expression with endometriosis are very contradictory [37,38,39,58]. There are studies where such a connection has not been found [58]; however, there are studies that show a direct correlation between the concentration/expression of SHBG and the risk of disease [37]. For example, the work of Panidis et al. revealed a higher level of SHBG in patients with endometriosis (compared with the control) and its decrease in the treatment of patients with danazol (the SHBG level in patients after treatment did not differ from those of healthy women) [37]. A decrease in the SHBG level (and an increase in free testosterone content) in endometriosis patients during treatment with danazol has been shown in other studies [83]. The Misao et al. study revealed an increased expression of *SHBG* mRNA in endometrioid tissue samples (pelvic endometriosis was studied) than in normal endometrium [38]. The authors point out that intercellular SHBG, which is excessively formed in endometrioid tissue, “protects” related estrogens from metabolic inactivation in the liver and makes them more accessible to endometrial cells, thereby causing an increased risk of endometriosis [38]. A later study by the same team of authors showed the dominant expression of a certain variant of *SHBG* mRNA associated with the peculiarities of exon VII splicing in ovarian endometriosis [39]. In accordance with the above experimental data, it can be noted that the risk value of SHBG-increasing genotype GG rs440837 (A > G) *ZBTB10* (OR = 1.91) identified in our study for endometriosis may be mediated by local (in endometrioid tissue) effects of higher levels of SHBG, which binds to estrogens and “protects” them from metabolic inactivation and thus makes them more accessible to endometrial cells (local hyperestrogenism is formed), thereby increasing the risk of endometriosis.

SHBG is a glycoprotein (the main place of SHBG formation is the liver), which has two analogous peptide chains containing specific sites for binding steroid hormones [48,73]. Its purpose in the organism is to bind/transport steroids (testosterone, estradiol, etc.) in plasma (due to the presence of specific sites) and thus regulate the biological activity of steroids (only steroids that are in the plasma in a free [unbound] state are biologically active) [48]. Based on the above, there is an obvious inverse relationship between, on the one hand, the SHBG level in plasma and, on the other hand, the content of free (bioactive) testosterone (primarily) and estrogens (secondly) in the female [48,73]. This is determined by the following circumstance: SHBG has a greater “sensitivity” to testosterone and a lesser “sensitivity” to estrogens, and as a result, a greater amount of testosterone compared to estradiol will be associated with SHBG (65% and 38%, respectively) [47,50]. If we take into account, at the same time, an approximately equal percentage in the amount of free (bioactive) forms of testosterone and estradiol in plasma (1–2% for testosterone and 2% for estradiol) [47,50], then we can make an assumption about a more pronounced effect of SHBG on the content/effects of testosterone in the organism compared to estradiol. So, an increase in the plasma SHBG content (including genetically determined) it will cause its more pronounced binding to testosterone than to estrogens, which will lead to a more significant decrease in free (bioactive) testosterone concentration (to a lesser extent estrogens) and eventually will have more serious phenotypically significant “consequences” in the female organism. The reverse-oriented “SHBG-bioactive testosterone” genetic relationship in women has been shown in a number of studies [46,49]: the indicators of “SHBG-bioactive testosterone” genetic correlations calculated in these works were very impressive and amounted to p_G_ = −0.60 [46] and r_g_ = −0.75 [49]. So, taking into account the above-mentioned results of the work, we can note that free (bioactive) testosterone-related effects may be at the heart of the established association of GWAS-important loci for SHBG with endometriosis.

Well, we found that SHBG-raising genotype GG rs440837 (A > G) *ZBTB10* causes an almost twofold increase in the endometriosis risk (OR = 1.91). An increase in the SHBG concentration, in turn, will determine a significant decrease in free testosterone in a woman [46,49], and this will eventually increase the risk of endometriosis, which we found in our study. Our results, to the fullest extent, correspond to the testosterone-driven concept of endometriosis development proposed in the review papers by Densdale & Crespi [40,41,42]. Densdale & Crespi postulate a hypothesis about the causal effect of low prenatal testosterone levels (persisting in the postnatal period) on an increased risk of developing endometriosis during a woman’s lifetime [40,41,42]. The works of these scientists indicate a pronounced effect on the hypothalamic-pituitary-gonadal system of the female fetus (in the prenatal period of its development) of low testosterone levels, which leads to its “reprogramming” and the formation of a certain endometriosis-predisposing pattern of the hormonal profile of these fetuses (reduced LH compared with FSH, increased SHBG, reduced systemic/ovarian testosterone, etc.) [40,41,42]. In their subsequent review article, Crespi et al. provided reasoned justifications for the positive correlation in a woman’s adult life of lower testosterone levels with the main endometriosis-related symptoms such as pelvic pain (several days a month) and increased pain sensitivity [84]. The authors note that the pathophysiological justification for the above correlations may be a link between low testosterone levels (on the one hand) and increased levels of the proinflammatory cytokine IL-1β, as well as reduced levels of β-endorphin (on the other hand) [84]. Crespi et al. point out that the low level of systemic/ovarian testosterone, formed in the prenatal period and maintained in the postnatal period, may be the basis for the formation of endometriosis and endometriosis-related symptoms (pain and sensitivity to it) [84]. The fundamental (genetic) basis of the prenatal/postnatal occurrence of low systemic/ovarian testosterone in a woman may be gene polymorphisms, which were established, among other things, in our present work.

A causal relationship between the genetic determination of low testosterone (both total and bioavailable) levels and endometriosis was found by McGrath I.M. et al., (UK Biobank data were used, the polygenic risk score was calculated, and Mendelian randomization was used) [45]. Conversely, Garitazelaia et al.’s work did not establish causal relationships between sex hormones (including SHBG) and endometriosis (GWAS from a Twins UK study [sex hormones] [54] and the FinnGen cohort [endometriosis] and MR were used) [61]. Our results fully correlate with the data obtained in the work of McGrath I.M. et al. [45] and differ from the results of Garitazelaia et al. [61]. The differences between our results (the association of the SHBG-raising genotype GG rs440837 (A > G) *ZBTB10* with an increased risk of endometriosis was revealed) and the data of Garitazelaia et al. (the MR method did not establish a link between the GWAS determinants of sex hormones and endometriosis [61]) may be due to the following points.

Firstly, Garitazelaia et al. analyzed a different list of polymorphisms associated with the level of sex hormones (8 SNPs were considered, which, according to GWAS data from Ruth et al. [54], were associated with such sex hormones (their indexes) as oestradiol [rs117585797 *ANO2*], dehydroepiandrosterone sulfate [rs148982377 *ZNF789*], follicle-stimulating hormone [rs11031005 *FSHB*], luteinizing hormone [rs11031002 *FSHB*], progesterone [rs112295236 *SLC22A10*, rs34670419 *ZKSCAN5*], SHBG [rs1641549 *TP53*]), and free androgen index [rs117145500 *CHD9*] compared to our work, which studied GWAS-significance for the level of nine SHBG polymorphisms [rs17496332 (A > G) *PRMT6*, rs780093 (C > T) *GCKR*, rs10454142 (T > C) *PPP1R21*, rs3779195 (T > A) *BAIAP2L1*, rs440837 (A > G) *ZBTB10*, rs7910927 (G > T) *JMJD1C*, rs8023580 (T > C) *NR2F2*, rs12150660 (G > T) *SHBG*, and rs4149056 (T > C) *SLCO1B1*]. Herewith, Garitazelaia et al. studied only one polymorphism associated with the SHBG level (rs1641549 *TP53*), whereas in our study, this SNP was not considered. Accordingly, different results can be expected for different panels of polymorphisms linked with different sex hormones under study.

Secondly, in the work of Garitazelaia et al., in which MR analysis did not demonstrate statistically significant causal relationships between the GWAS-impact determinant of the sex hormone levels and endometriosis, pleiotropic genetic associations of two loci (rs11031002; rs11031005) of the *FSHB* gene with endometriosis and sex hormone contents were registered (and this, as the authors themselves note, came as a surprise to them!). Also, two SNPs (rs11031005; rs11031006) in this region of *FSHB* demonstrated statistically substantial pleiotropic associations mediating endometriosis and related features (age of menarche and menopause, and duration of the menstrual cycle) [61]. Thus, even within the framework of a single large-scale MR analysis of GWAS data, there is a serious discrepancy in the study results: the absence of associations for the totality of the analyzed 8 SNPs and the presence of associations for two separate loci! This data may indicate that the effects of individual polymorphisms may not “coincide” with the total effects of a group of polymorphisms, and further experimental genetic studies in the field of endometriosis are needed with a detailed analysis of both the effects of individual loci and an assessment of the total effect of a group of polymorphisms (causal relationships) in the development of the disease.

Quite importantly, in our earlier study by Golovchenko et al. [60], devoted to the relationship of GWAS-impact SNP of sex hormones (this panel of 8 loci taken from a GWAS by Ruth et al. [54]) with endometriosis, we studied (the work was performed on the same sample of patients): first, the disorder-protective value of total/free testosterone-boosting genetic variant C rs11031005 *FSHB*; and secondly, involvement in predisposition to the disease (as part of intergenic interactions) of polymorphisms, GWAS-impact on the circulation level of SHBG (rs1641549 *TP53* [54], rs727428 *SHBG* [52]), free [bioavailable] testosterone (rs112295236 *SLC22A10* [55], rs727428 *SHBG* [55]), DHEAS (rs34670419 *ZKSCAN5* [85]) [60]. The correlation of testosterone levels in patients with endometriosis with polymorphisms rs148982377 *ZNF789* and rs34670419 *ZKSCAN5* was shown [86].

It should be noted that despite large-scale genetic studies of endometriosis (numerous associative studies and more than 25 GWAS [10,11,12,13,14,15,16,17,18,19,20,21,22,23,24,25,26,27,28,29,30,31,32,33,34,35,36]), data on specific genetic factors are currently known for only a small part (approximately 5%) of the variability of the disease [16,30]. This suggests that the genetic basis of endometriosis remains largely unclear, since the currently known SNPs/genes associated with endometriosis (involved in the production of sex (steroid) hormones (*SYNE1*, *FN1*, *FSHB*, *ESR1*, *CCDC170*, etc.) [16,60], pain sensitivity (*MLLT10*, *BMF/SRP14*, *NGF*, *GDAP1*, *BSN*, etc.) [30], fat distribution (*KIFAP3*, *CAB39L*, *WNT4*, *GRB14*, etc.) [69], affecting the structure of chromatin (*TRA2A*, *GREB1*, *VEZT*, *BMF*, *SRP14*, etc.) [30], inflammatory reactions, uterine development, oncogenesis, etc. [30], explain only about one twentieth of the total variability and one tenth of the genetic variability of the disease. This fact underscores the need to continue further active research on endometriosis in order to identify other genetic factors that determine the development of the disease, which currently, despite the serious efforts made by the scientific community in this direction, remain unknown.

This work has a number of limitations, among which the following can be noted. Firstly, our results were obtained for one ethnic group (the Europeans of Central Russia) and can only be used as a guideline for other ethnic groups. Further research in this area is needed to confirm the patterns identified in this work in other ethnic groups. Secondly, due to the small number of women in the control group with infertility, we were unable to conduct a representative comparative analysis of the genetic characteristics of patients and controls with infertility according to the considered SHBG-significant polymorphisms.

## 4. Materials and Methods

### 4.1. Study Subjects

When planning/conducting this study, the requirements/recommendations of the “Ethics Committee” of the Belgorod State National Research University were taken into account: the procedure for implementing each stage of the study (collection of clinical/anamnestic/biological data, laboratory [genetic] testing, etc.) was considered in detail/approved at meetings of this committee; all participants in our study confirmed their consent in writing to their involvement in it. The study was conducted on a total sample of 1368 women (395 endometriosis; 973 endometriosis free [controls]). The sample was ethnically/geographically “homogeneous” and included only Caucasian (Russian) women who were born/lived in Central Russia [87,88,89]. Individuals with oncological pathology of the female reproductive system (pelvic organs, mammary gland), and severe disorder of the vital organs and autoimmune system, were excluded from the study (both endometriosis and controls) [60].

The diagnosis of endometriosis was carried out on the basis of rASRM criteria [90] by certified doctors of the gynecology and assisted reproductive technologies departments at the Perinatal Center, and included mandatory morphological verification of the endometrioid tissue detected during laparotomy/laparoscopy. Among patients (*n* = 395), stage I of the disorder was detected in 35.90%, stage II in 53.98%, and stage III/IV in 10.12%. The absence of well-known clinical symptoms of endometriosis in women (infertility, chronic pelvic pain, and others), as well as the reproductive system (pelvic organ) disorders, allowed them to be included in the control group (endometriosis-free).

### 4.2. Experimental Analysis of the DNA (Selection and Genotyping of SNP)

DNA samples (concentrations ranging from 10 to 20 ng/mL with a sufficient level of purity [parameter “260 nm/280 nm” was in the range from 1.7 to 2.0] [91] according to the results of testing on NanoDrop 2000 (Thermo Fisher Scientific Inc., Waltham, MA, USA)) were genotyped on a CFX96 amplifier [92,93]. The polymorphisms panel for genotyping included 9 SNPs that were GWAS-validated for SHBG [51,53,55,56,57,94] (Table S9) and had forecasted functionality (Table S3). So, loci such as rs17496332 (A > G) *PRMT6*, rs780093 (C > T) *GCKR*, rs10454142 (T > C) *PPP1R21*, rs3779195 (T > A) *BAIAP2L1*, rs440837 (A > G) *ZBTB10*, rs7910927 (G > T) *JMJD1C*, rs8023580 (T > C) *NR2F2*, rs12150660 (G > T) *SHBG*, and rs4149056 (T > C) *SLCO1B1* were detected in our work (previously, this panel of loci was “tested” and showed its “effectiveness” in the genetic study of breast cancer, endometrial hyperplasia, and uterine fibroid [62,78,95,96,97,98,99]).

The “call rate” indicator was used to control the quality of experimentally obtained genetic data: DNA samples and SNPs with a “call rate” of >90% were considered to have “completed” quality control and were included in the study. All the DNA samples studied and the SNPs considered had a “call rate” > 90% (for the studied SNPs, the “call rate” in the endometriosis group was 92.41% [rs440837 (A > G) *ZBTB10*]—99.24% [rs17496332 (A > G) *PRMT6*], and in the control group, 90.85% [rs4149056 (T > C) *SLCO1B1*]—96.40% [rs12150660 (G > T) *SHBG*] (Table S1); the total genotyping rate in the studied case/control individuals was 94.76%). The “blind re-genotyping” method (for this purpose, repeated studies were conducted for ≈5% of the studied DNA samples), which we additionally used to control the quality of the obtained genetic data [100], showed complete coincidence in the results obtained (certain genotypes) for 99% of the “blind re-genotyped” samples, which indicates the acceptable quality of the genetic analysis performed.

### 4.3. Statistical Analysis of Experimental Genetic Data (SNP and Multi-SNPs Association Analysis)

A mandatory “preparatory” stage before evaluating “endometriosis–SNP” associations was a comparison of the observed and expected (when performing the Hardy–Weinberg equilibrium [HWE]) genotype frequencies for each examined polymorphism for both endometriosis and endometriosis-free, and when these frequencies corresponded, the locus was considered “suitable” for inclusion in the associative analysis.

To assess the “endometriosis–SNP” relationship (calculation of relationship indicators such as the odds ratio [OR] and its 95% confidence interval [95% CI] was performed [101])), the method of logistic regression was employed, and four “basic” models of the reference/alternative allele interactions at the studied polymorphisms (additive//recessive//dominant//allelic [102]) were considered. All calculations were performed in the PLINK (Java-compatible version) software product, which is most widely utilized for solution goals like this [103], when adjusted for confounders (as mentioned above [Table 1], the confounders were menstrual cycle duration, parity, the anamnesis presence of medical abortions (as well as their number), infertility, surgical procedures (laparoscopy; laparotomy) on pelvic organs, and burdened heredity), with multiple comparisons (an adaptive permutation test was used, as a result of which the p_perm_ value was assessed [104]) and mandatory determination of the power of the identified associative relationships (the Quanto program was utilized [105]). A value of p_perm_ less than 0.05 with a power of at least 80% was the basis for the identification of statistically significant/justified “endometriosis–SNP” associations [106].

To study the contribution of multi-SNPs (non-allelic variant interlocus interactions) of SHBG-impact genes to susceptibility to endometriosis, the MB-MDR method [107] and the software of the same name for the R package were exchanged. When evaluating associations, the necessary confounders were taken into account (menstrual cycle duration, parity, the anamnesis presence of medical abortions (as well as their number), infertility, surgical procedures (laparoscopy and laparotomy) on pelvic organs, burdened heredity) and permutation testing was performed (the number of permutations performed was 1000). For permutation validation, one of the most significant “endometriosis-multi-SNP” models was selected (they have the highest values of both Wald statistics and the level of statistical significance [*p*]) of each considered level. At the same time, this procedure (permutation validation) included only those models that exceeded a certain “threshold level” in terms of statistical significance, calculated by us taking into account the Bonferroni correction (the generally accepted statistically significant parameter “*p*” equal to 0.05 was adjusted for the number of all possible combinations of the nine studied loci with their different-level combinations) and which made up the following values: two (p_threshold_ = 1.39 × 10^−3^ [0.05/36]), three (p_threshold_ = 5.95 × 10^−4^ [0.05/84]), four (p_threshold_ = 3.97 × 10^−4^ [0.05/126]) and five (p_threshold_ = 3.97 × 10^−4^ [0.05/126]) locus interactions. In the end, p_perm_ values not exceeding 0.01/0.001 for two-locus/three-and-higher locus models, respectively, were considered reliable. To visualize the endometriosis-significant effects of SHBG-impact loci (orientation and percentage of contribution (entropy) to the disease), the MDR software package was used [108].

### 4.4. Analysis of Forecasted Functionality at Endometriosis-Associated Polymorphisms (In Silico Study)

An essential final part of our work was the assessment of the potential functionality of endometriosis-associated loci and their LD SNPs (these loci were distinguished at the value of the r^2^ parameter from 0.80 to 1.00) [109,110] based on materials presented in such well-reputed bioinformatic/experimental genetic databases [111,112] as HaploReg (v.4.2, accessed 11 October 2024) [113]; GTExportal (accessed 1 October 2024) [114]; STRING (accessed 16 October 2024) [115].

## 5. Conclusions

SHBG-raising genotype GG rs440837 (A > G) *ZBTB10* and GWAS-substantial intergenic interactions affect the level of seven SHBG SNPs and increase the risk of endometriosis in Caucasian women of Russia.

## Figures and Tables

**Table 1:** Characteristics of participants from the case and control groups.

Parameters	Cases (*n* = 395) $\bar{X}$ ± SD/% (*n*)	Controls (*n* = 973) $\bar{X}$ ± SD/% (*n*)	*p*
Age, years	39.75 ± 9.01	40.71 ± 8.60	>0.05
Height, m	1.65 ± 0.06	1.65 ± 0.06	>0.05
Weight, kg	72.65 ± 14.38	72.47 ± 13.36	>0.05
BMI, kg/m^2^	26.63 ± 5.31	26.65 ± 4.60	>0.05
Proportion of the participants by relative BMI, % (*n*):			
Underweight (<18.50)	4.30 (17)	1.13 (11)	
Normal weight (18.50–24.99)	37.72 (149)	42.55 (414)	>0.05
Overweight (25.00–29.99)	31.65 (125)	30.32 (295)	
Obese (>30.00)	26.33 (104)	26.00 (253)	
Family history of endometriosis (yes)	6.07 (24)	1.95 (19)	**<0.001**
Married	82.53 (326)	85.82 (835)	>0.05
Smoking (yes)	18.22 (72)	17.47 (170)	>0.05
Drinking alcohol (≥7 drinks per week)	4.05 (16)	3.08 (30)	>0.05
History of pelvic surgery (laparoscopy and/or laparotomy)	15.19 (60)	9.76 (95)	**<0.01**
Oral contraceptive use	8.10 (32)	9.76 (95)	>0.05
Age at menarche and menstrual cycle			
Age at menarche, years	13.29 ± 1.27	13.26 ± 1.25	>0.05
Proportion of the participants by relative age at menarche, % (*n*)			
Early (<12 years)	6.36 (25)	6.47 (63)	
Average (12–14 years)	81.17 (319)	80.16 (780)	>0.05
Late (>14 years)	12.47 (49)	13.36 (130)	
Duration of bleeding menstrual (mean, days)	5.13 ± 1.56	4.95 ± 0.93	>0.05
Menstrual cycle length (mean, days)	27.66 ± 2.28	28.16 ± 2.25	**<0.001**
Reproductive characteristic			
Age at first birth (mean. years)	21.25 ± 3.04	21.70 ± 3.48	>0.05
Gravidity (mean)	2.60 ± 2.31	2.46 ± 1.56	>0.05
No. of births (mean)	1.07 ± 0.97	1.51 ± 0.67	**<0.001**
No. of spontaneous abortions (mean)	0.21 ± 0.61	0.24 ± 0.51	>0.05
No. of induced abortions (mean)	1.25 ± 1.61	0.67 ± 0.99	**<0.001**
No. of induced abortions:			
0	46.58 (184)	58.99 (574)	
1	17.22 (68)	23.54 (229)	
2	19.24 (76)	10.48 (102)	
3	8.61 (34)	5.45 (53)	**<0.001**
≥4	8.35 (33)	1.54 (15)	
History of infertility	32.42 (132)	5.24 (51)	**<0.001**
Gynecological pathologies			
Uterine leiomyoma	52.40 (207)	-	**-**
Endometrial hyperplasia	46.33 (183)	-	**-**
Adenomyosis	43.04 (170)	-	**-**

*p* values < 0.05 are shown in bold.

**Table 2:** Associations of the SHBG-significant gene polymorphisms with endometriosis.

SNP	Gene	Minor Allele	*n*	Allelic Model				Additive Model				Dominant Model				Recessive Model			
				OR	95%CI		*p*	OR	95%CI		*p*	OR	95%CI		P	OR	95%CI		*p*
					**L95**	**U95**			**L95**	**U95**			**L95**	**U95**			**L95**	**U95**	
rs17496332	*PRMT6*	G	1292	1.01	0.84	1.20	0.941	1.03	0.86	1.23	0.761	1.01	0.78	1.30	0.959	1.01	0.77	1.57	0.598
rs780093	*GCKR*	T	1310	0.98	0.83	1.17	0.828	0.97	0.81	1.16	0.727	0.97	0.75	1.26	0.836	0.93	0.66	1.33	0.699
rs10454142	*PPP1R21*	C	1291	0.87	0.72	1.05	0.153	0.91	0.74	1.10	0.321	0.84	0.65	1.08	0.170	1.03	0.67	1.58	0.907
rs3779195	*BAIAP2L1*	A	1290	1.02	0.82	1.27	0.870	1.02	0.81	1.29	0.860	1.12	0.86	1.47	0.405	0.49	0.20	1.15	0.100
rs440837	*ZBTB10*	G	1282	1.01	0.83	1.24	0.915	1.02	0.82	1.26	0.868	0.91	0.70	1.18	0.474	**1.91**	**1.13**	**2.98**	**0.023**
rs7910927	*JMJD1C*	T	1311	0.89	0.75	1.05	0.164	0.89	0.75	1.07	0.208	0.80	0.61	1.07	0.128	0.93	0.69	1.25	0.610
rs4149056	*SLCO1B1*	C	1264	0.93	0.76	1.14	0.490	0.94	0.76	1.17	0.601	0.91	0.70	1.18	0.472	1.06	0.60	1.87	0.852
rs8023580	*NR2F2*	C	1304	0.99	0.82	1.19	0.905	1.03	0.85	1.26	0.763	1.13	0.87	1.45	0.355	0.78	0.47	1.27	0.316
rs12150660	*SHBG*	T	1323	0.91	0.74	1.10	0.327	0.88	0.72	1.09	0.243	0.92	0.71	1.18	0.495	0.64	0.36	1.12	0.120

All results were obtained after adjustment for covariates; OR, odds ratio; 95% CI, 95% confidence interval; *p* values < 0.05 are shown in bold; the additive genetic model evaluates how the phenotypic effect will change with an increase in the “dose” of the recessive allele in the genotype (all genotypes are compared; for example, TT vs. TC vs. CC, where C is the minor allele); the dominant model assumes that the minor allele exhibits a dominant effect, and the major allele is recessive (CC/TC vs. TT, where C is the minor allele); the recessive model assumes that the minor allele has a recessive effect, and the major allele is dominant (CC vs. TC/TT, where C is the minor allele); the allele model assumes a comparison of genotypes having the recessive allele/alleles with the genotype without these alleles (has the dominant allele) (C vs. T, where C is the minor allele).

**Table 3:** SNP × SNP interactions of SHBG-significant genes associated with endometriosis.

N	SNP × SNP Interaction Models	NH	*beta*H	WH	NL	*beta*L	WL	p_perm_
Two-order interaction models (*p* = 1.25 × 10^−3^)								
1	rs440837 *ZBTB10*-rs3779195 *BAIAP2L1*	1	0.412	4.07	1	−0.513	10.41	0.008
Three-order interaction models (*p* = 8.33 × 10^−6^)								
2	rs8023580 *NR2F2*-rs440837 *ZBTB10*-rs3779195 *BAIAP2L1*	5	0.612	19.86	2	−0.495	9.20	0.001
Four-order interaction models (*p* = 9.65 × 10^−8^)								
3	rs440837 *ZBTB10*-rs10454142 *PPP1R21*-rs780093 *GCKR*-rs17496332 *PRMT6*	7	1.193	28.44	2	−1.118	6.81	0.001
Five-order interaction models (*p* = 1.95 × 10^−11^)								
4	rs8023580 *NR2F2*-rs7910927 *JMJD1C*-rs440837 *ZBTB10*-rs3779195 *BAIAP2L1*- rs780093 *GCKR*	9	1.550	45.02	1	−1.384	4.67	0.001

The results were obtained using the MB-MDR method with adjustment for covariates; NH, number of significant high risk genotypes in the interaction; *beta*H, regression coefficient for high risk exposition in the step2 analysis; WH, Wald statistic for high risk category; NL, number of significant low risk genotypes in the interaction; *beta*L, regression coefficient for low risk exposition in the step2 analysis; WL, Wald statistic for low risk category; p_perm_, permutation *p*-value for the interaction model (1.000 permutations).

## Data Availability

The authors declare that the data supporting the findings of this study are available within the paper and its Supplementary Tables. The raw data used in this study can be obtained from the corresponding author upon reasonable request.

## Supplementary Materials

The following supporting information can be downloaded at: https://www.mdpi.com/article/10.3390/ijms262311630/s1.

## Abbreviations

The following abbreviations are used in this manuscript:

SHBG	Sex hormone-binding globulin
SHBG_level_	Sex hormone-binding globulin level
SNP	Single-nucleotide polymorphism
GWAS	Genome-wide association studies
BMI	Body mass index
DNA	Deoxyribonucleic acid
LD	Linkage disequilibrium
TFs	Transcription factors
