# Supplementary material for: Gene Polymorphisms Determining Sex Hormone-Binding Globulin Levels and Endometriosis Risk
Source: Int J Mol Sci. 2025 Nov 30;26(23):11630. doi: 10.3390/ijms262311630 (PMC12691900; doi:10.3390/ijms262311630)
Supplement: Supplementary file 1 [file ijms-26-11630-s001.zip › !Suppl table S2.pdf]

Supplementary table 2

Genotype combinations associated with endometriosis *

Model	N	Genotype combinations	*beta*	P	Risk, High /Low
Two-order interaction models					
1	1	rs440837-AG *ZBTB10*-rs3779195-TT *BAIAP2L1*	-0.513	0.001	L
	2	rs440837-AG *ZBTB10*-rs3779195-TA *BAIAP2L1*	0.411	0.044	H
Three-order interaction models					
2	1	rs8023580-TC *NR2F2*-rs440837-AA *ZBTB10*-rs3779195-TT *BAIAP2L1*	0.358	0.029	H
	2	rs8023580-TT *NR2F2*-rs440837-AG *ZBTB10*-rs3779195-TT *BAIAP2L1*	-0.429	0.032	L
	3	rs8023580-TT *NR2F2*-rs440837-GG *ZBTB10*-rs3779195-TT *BAIAP2L1*	0.913	0.037	H
	4	rs8023580-TC *NR2F2*-rs440837-AG *ZBTB10*-rs3779195-TA *BAIAP2L1*	0.629	0.042	H
Four-order interaction models					
3	1	rs440837-AA *ZBTB10*-rs10454142-CC *PPP1R21*-rs780093-CC *GCKR*-rs17496332-AA *PRMT6*	1.362	0.045	H
	2	rs440837-AA *ZBTB10*-rs10454142-TT *PPP1R21*-rs780093-TT *GCKR*-rs17496332-AA *PRMT6*	0.982	0.018	H
	3	rs440837-AA *ZBTB10*-rs10454142-TT *PPP1R21*-rs780093-CT *GCKR*-rs17496332-GG *PRMT6*	0.975	0.016	H
Five-order interaction models					
4	1	rs8023580-TC *NR2F2*-rs7910927-GG *JMJD1C*-rs440837-AA *ZBTB10*-rs3779195-TT *BAIAP2L1*- rs780093-CT *GCKR*	1.033	0.019	H
	2	rs8023580-TT *NR2F2*-rs7910927-TT *JMJD1C*-rs440837-AA *ZBTB10*-rs3779195-TT *BAIAP2L1*- rs780093-CT *GCKR*	-1.384	0.031	L
	3	rs8023580-TT *NR2F2*-rs7910927-TT *JMJD1C*-rs440837-AA *ZBTB10*-rs3779195-TA *BAIAP2L1*- rs780093-CT *GCKR*	1.107	0.047	H
	4	rs8023580-TT *NR2F2*-rs7910927-GG *JMJD1C*-rs440837-AG *ZBTB10*-rs3779195-TA *BAIAP2L1*- rs780093-CT *GCKR*	2.887	0.008	H
	5	rs8023580-TC *NR2F2*-rs7910927-GT *JMJD1C*-rs440837-AG *ZBTB10*-rs3779195-TA *BAIAP2L1*- rs780093-CT *GCKR*	1.698	0.006	H
	6	rs8023580-TT *NR2F2*-rs7910927-TT *JMJD1C*-rs440837-AA *ZBTB10*-rs3779195-TT *BAIAP2L1*- rs780093-TT *GCKR*	1.371	0.041	H

* Genotype combinations are derived from the interaction models obtained by the MB-MDR method and described in tables 1
