# Supplementary material for: Gene Polymorphisms Determining Sex Hormone-Binding Globulin Levels and Endometriosis Risk
Source: Int J Mol Sci. 2025 Nov 30;26(23):11630. doi: 10.3390/ijms262311630 (PMC12691900; doi:10.3390/ijms262311630)
Supplement: Supplementary file 1 [file ijms-26-11630-s001.zip › !Suppl table S9.pdf]

**Supplementary Table S9.** The GWAS data about associations of the studied candidate gene polymorphisms with the circulating SHBG and other sex hormone concentrations.

| SNP, gene | Chromosome position (hg38) | Phenotype | Association (significance) (affected allele) | Reference |
|---|---|---|---|---|
| rs17496332 *PRMT6* | 1p13.3 (107003753) | SHBG | β=-0.028 ($p=1\times10^{-11}$) (A) | [53] |
| rs780093 *GCKR* | 2p23.3 (27519736) | SHBG | β=-0.032 ($p=2\times10^{-16}$) (T) | [53] |
| rs10454142 *FOXN2* | 2p16.3 (48419260) | SHBG | β=0.026 ($p=1\times10^{-7}$) (T) | [53] |
| rs3779195 *BAIAP2L1* | 7q21.3 (98364050) | SHBG | β=-0.033 ($p=3\times10^{-8}$) (A) | [53] |
| | | SHBG (women, pre-menopause) | β=-2.41 ($p=9\times10^{-09}$) (A) | [57] |
| rs440837 *ZBTB10* | 8q21.13 (80549739) | SHBG | β=-0.030 ($p=3\times10^{-9}$) (A) | [53] |
| | | SHBG (women, post-menopause) | β=1.43 ($p=1\times10^{-12}$) (G) | [57] |
| | | SHBG (men) | β=0.57 ($p=8\times10^{-9}$) (G) | [57] |
| rs7910927 *JMJD1C* | 10q21.3 (63379150) | SHBG | β=-0.048 ($p=6\times10^{-35}$) (T) | [53] |
| rs4149056 *SLCO1B1* | 12p12.1 (21178615) | SHBG | β=0.029 ($p=2\times10^{-8}$) (T) | [53] |
| | | low testosterone levels (men) | OR=1.14 ($p=7\times10^{-16}$) (C) | [94] |
| | | testosterone (women) | β=0.028 ($p=5\times10^{-10}$) (C) | [56] |
| | | SHBG (women) | β=-0.065 ($p =5\times10^{-48}$) (C) | [56] |
| | | SHBG (women, pre-menopause) | β=-0.062 ($p=8\times10^{-11}$) (C) | [56] |
| | | SHBG (women, post-menopause) | β=-0.079 ($p=7\times10^{-34}$) (C) | [56] |
| | | bioavailable testosterone (women) | β=0.02 ($p=2\times10^{-16}$) (C) | [57] |
| | | SHBG (men) | β=-1.23 ($p=7\times10^{-29}$) (C) | [57] |
| | | SHBG (women) | β=0.030 ($p=1\times10^{-73}$) (T) | [55] |
| | | SHBG (men) | β=0.032 ($p=6\times10^{-99}$) (T) | [55] |
| | | total testosterone (women) | β=-0.029 ($p=1\times10^{-14}$) (T) | [55] |
| | | bioavailable | β=-0.043 ($p=3\times10^{-35}$) (T) | [55] |

| | | | | |
|---|---|---|---|---|
| | | testosterone (women) | | |
| | | total testosterone (men) | β=0.054 ($p=1\times10^{-39}$) (T) | [55] |
| rs8023580 *PPP1R21* | 15q26.2 (96165062) | SHBG | β=-0.03 ($p=8\times10^{-12}$) (T) | [53] |
| | | low testosterone levels (men) | OR=1.13 ($p=1\times10^{-19}$) (T) | [94] |
| rs12150660 *SHBG* | 17p13.1 (7618597) | SHBG | β=0.103 ($p=2\times10^{-106}$) (T) | [53] |
| | | SHBG (women) | β=6.14 ($p=1\times10^{-300}$) (T) | [57] |
| | | SHBG (men) | β=3.9 ($p=2\times10^{-75}$) (T) | [51] |
| | | total testosterone (men) | β=31.8 ($p=1\times10^{-41}$) (T) | [51] |
