# Supplementary material for: Gene Polymorphisms Determining Sex Hormone-Binding Globulin Levels and Endometriosis Risk
Source: Int J Mol Sci. 2025 Nov 30;26(23):11630. doi: 10.3390/ijms262311630 (PMC12691900; doi:10.3390/ijms262311630)
Supplement: Supplementary file 1 [file ijms-26-11630-s001.zip › 30.10.25 !Suppl table S1.pdf]

Supplementary Table S1. The allele and genotype frequencies of the studied SNPs in the endometriosis and control groups.

Chr	SNP	Gene	Minor allele	Major allele	Minor allele frequency	Number of the studied chromosomes (call rate,%)	Genotype distribution*	H_o	H_e	P_{HWE}
Endometriosis (n=395)										
1	rs17496332	*PRMT6*	G	A	0.369	784 (99.24)	56/170/156	0.445	0.466	0.381
2	rs780093	*GCKR*	T	C	0.395	758 (95.95)	56/187/136	0.493	0.478	0.591
2	rs10454142	*PPP1R21*	C	T	0.291	736 (93.16)	34/146/188	0.397	0.412	0.451
7	rs3779195	*BAIAP2L1*	A	T	0.178	746 (94.43)	7/119/247	0.319	0.293	0.111
8	rs440837	*ZBTB10*	G	A	0.240	730 (92.41)	27/121/217	0.332	0.365	0.085
10	rs7910927	*JMJD1C*	T	G	0.466	760 (96.20)	84/186/110	0.490	0.498	0.758
12	rs4149056	*SLCO1B1*	C	T	0.217	760 (96.20)	19/127/234	0.334	0.340	0.763
15	rs8023580	*NR2F2*	C	T	0.283	746 (94.43)	23/165/185	0.442	0.406	0.097
17	rs12150660	*SHBG*	T	G	0.233	770 (97.47)	17/145/223	0.377	0.357	0.319
Control (n=973)										
1	rs17496332	*PRMT6*	G	A	0.368	1820 (93.53)	128/413/369	0.454	0.465	0.476
2	rs780093	*GCKR*	T	C	0.399	1862 (95.68)	147/449/335	0.482	0.480	0.891
2	rs10454142	*PPP1R21*	C	T	0.320	1846 (94.86)	97/396/430	0.429	0.435	0.705
7	rs3779195	*BAIAP2L1*	A	T	0.176	1834 (94.24)	37/248/632	0.270	0.290	0.052
8	rs440837	*ZBTB10*	G	A	0.238	1834 (94.24)	42/352/523	0.384	0.362	0.083
10	rs7910927	*JMJD1C*	T	G	0.496	1862 (95.68)	227/469/235	0.504	0.500	0.844
12	rs4149056	*SLCO1B1*	C	T	0.230	1768 (90.85)	48/310/526	0.351	0.354	0.776
15	rs8023580	*NR2F2*	C	T	0.285	1862 (95.68)	88/355/488	0.381	0.408	0.053
17	rs12150660	*SHBG*	T	G	0.251	1876 (96.40)	67/336/535	0.358	0.376	0.164

Note: * minor allele homozygotes / heterozygotes / major allele homozygotes
